# Endoscopic Approach in the Diagnosis of Gastrointestinal Acute Graft Versus Host Disease in Children

**DOI:** 10.1097/PG9.0000000000000163

**Published:** 2022-01-24

**Authors:** Maura Faraci, Stefano Giardino, Annalisa Madeo, Cristina Coccia, Serena Arrigo, Filomena Pierri, Paolo Gandullia

**Affiliations:** From the * Hematopoetic Stem Cell Transplant Unit, Hematology-Oncology Department, IRCSS-Istituto G. Gaslini, Genova, Italy; † Gastroenterology and Digestive Endoscopy Unit, IRCSS—Istituto G. Gaslini, Genova, Italy; ‡ Department of Pathology, IRCSS-Istituto G. Gaslini, Genova, Italy.

**Keywords:** hematopoietic stem cell transplantation, bowel, GvHD

## Abstract

The objectives of this study were to describe clinical, histological, and endoscopic findings in children undergoing gastrointestinal (GI) endoscopy for symptoms of digestive acute graft-versus-host disease (a-GvHD), to evaluate the rate of steroid-resistant GvHD and transplant-related mortality, and to describe the feasibility and safety of the endoscopic procedure.

**Methods:**

This was a retrospective study conducted, at the IRCSS Istituto G. Gaslini in Genova, Italy, in 26 children undergoing upper or lower GI endoscopy after allogeneic hematopoietic stem cell transplantation between 2000 and 2017.

**Results:**

Histology confirmed a diagnosis of a-GvHD in 73% of patients; it was frequently associated with steroid-resistant a-GvHD (*P* = 0.001) and with an increased transplant-related mortality. Additionally, one patient developed duodenal hematoma after endoscopy for a high-grade GI a-GvHD.

**Conclusions:**

In our experience, the endoscopic approach in the diagnosis of GI a-GvHD in children was feasible and safe. Furthermore, the histological diagnosis of GI a-GvHD was associated with an increased risk of steroid-resistant GvHD and with high transplant-related mortality.

**What is Known/What is New/Translational Impact**

**What Is Known**

- Endoscopy and histological evaluation of GI a-GvHD may help in its diagnosis.

**What Is New**

- The sensitivity of histological evaluation in confirming the clinical diagnosis of a-GvHD was 73.1%, and it was higher when endoscopy was performed in the lower gastrointestinal tract (81.8%) than in the upper GI tract (57.1%).
- The histological confirmation of any grade of GI a-GvHD was associated with a significantly higher rate of steroid resistance (*P* = 0.028) and increased transplant-related mortality (TRM) (28.9%) when compared with a negative histology.

## INTRODUCTION

Graft versus host disease (GvHD) is the most important complication of allogeneic stem cell transplantation (allo-SCT). Acute GvHD (a-GvHD) may involve the gastrointestinal (GI) tract with clinical manifestations similar to other complications that may occur after SCT, such as viral, bacterial, or protozoan infections, as well as adverse effects of chemotherapy, immunosuppressive drugs such as mycophenolate mofetil (MMF), and antibiotics. The diagnosis and grading of a-GvHD is based on the Glucksberg criteria (1), and histological evaluation of the rectal mucosal biopsy may be helpful in confirming the diagnosis. However, the role of GI endoscopy in this setting is still debated (2,3). An increase in epithelial cell apoptosis in the proliferative zone of crypts, degeneration and necrosis of crypts, acute neutrophil infiltration, ulceration, and mucosal loss represent the histological features of GI a-GvHD. These findings may also be associated with other transplant-related gastrointestinal complications (e.g., MMF treatment-related complications and viral infections (4–9)). Although universal agreement on a GI a-GvHD histological grading system is lacking (9), histological findings of GI a-GvHD may be graded using a 4-point validated scale (adapted from Cruz-Correa et al. (10)). Currently, only a few studies have been published on GI endoscopy in children (5–8).

The aims of this retrospective study were to describe the clinical, histological, and endoscopic findings in children undergoing GI endoscopy for symptoms of GI a-GvHD, to evaluate the rate of steroid-resistant GvHD and transplant-related mortality, and to describe the feasibility and safety of the endoscopic procedure in these patients.

## METHODS

This was an observational retrospective study conducted in all children who underwent GI endoscopy for suspected a-GvHD after allo-SCT at the IRCSS Istituto G. Gaslini in Genova, Italy, between January 2000 and December 2017. During the study period, 348 allo-SCTs were performed in children with malignant or nonmalignant diseases. The diagnosis and grading of a-GvHD were based on the Glucksberg clinical criteria (1).

According to local guidelines, GI endoscopic and histological evaluations are indicated in children with (a) clinical signs of GI a-GvHD in the absence of any other organ involvement, to allow a differential diagnosis with other gastrointestinal complications, such as viral infections; (b) persistent GI symptoms after partial resolution of multiorgan a-GvHD. For each patient, the choice to perform endoscopic procedures to examine the upper GI tract (esophagogastroduodenoscopy [EGD]) or the lower GI tract (pan-colonoscopy [PC], flexible sigmoidoscopy [FS], or rectal suction biopsy) was based on the main presenting GI symptoms and on the evaluation of the risks associated with the procedure in each patient. In our center, before 2005, rectal suction biopsy was frequently used to diagnose GI-GvHD. However, after 2005, FS with rectal mucosa evaluation became the most common diagnostic approach. Endoscopic findings were evaluated according to the “Freiburg Criteria” (11). The preconditions needed to perform the procedures included a platelet count above 50.000/mmc and normality of coagulation values.

In all the patients, microbiological examinations of stools were performed at the onset of diarrhea, and polymerase chain reaction (PCR) was performed on blood and mucosal biopsies to detect viral infections. The mucosal biopsies obtained by the endoscopies were reviewed by the same pathologist. Histological evaluation was based on a 4-point validated scale of GI a-GvHD (adapted from Cruz-Correa et al (10)) including: grade I, increased crypt apoptosis; grade II, apoptosis with crypt abscess; grade III, individual crypt necrosis; grade IV, total denudation of areas of mucosa.

Either the transplanted patients or their guardians signed a consent form, allowing the use of their data for clinical research purposes. The Liguria Regional Ethics Committee approved data collection from the medical records of the patients admitted to our department for retrospective studies (360REG2014).

### Statistical Analysis

Descriptive statistics were reported in terms of absolute frequencies and percentage data, and the Pearson’s chi-square test or Fisher’s exact test, when appropriate, were used to compare proportions. The cumulative overall survival (OS) and cumulative transplant-related mortality (TRM) were calculated using the Kaplan-Meier method. The log-rank test was used to compare the survival distribution among groups. All tests were two-tailed, and a *P* < 0.05 was considered statistically significant.

## RESULTS

In our cohort of 348 allo-SCTs performed between 2000 and 2017, GI tract involvement was clinically diagnosed in 50 children (24%), and GI endoscopies were performed in 26 of those 50 (52%). The characteristics of these 26 patients and the cohort of patients who did not receive endoscopic approaches are summarized in Table 1, while the clinical grade of GvHD and therapies administered to each patient are detailed in Table 2.

**TABLE 1.** Clinical and transplant features of 26 patients with clinical diagnosis of gastrointestinal acute GvHD submitted gastrointestinal endoscopy and biopsies

	Endoscopy group = 26	Not endoscopy group = 322
Diagnosis, n (%)		
Acute leukemia—Myelodisplasia	14 (54)	147 (48%)
Bone marrow failure	7 (27)	51 (19%)
Congenital immunodeficiency	3 (11)	28 (29)
Hemoglobinopathy	2 (8)	11 (4)
**Age at transplant**, median, y (range)	9.5 (0.5–16.9)	8.15 (0.38–21)
**Gender**, n (%)		
Male	17(65)	171 (54)
Female	9(35)	151 (46)
**Type of donor**, n (%)		
Alternative	21 (80)	95 (29)
Related	4 (16)	83 (26)
Haploidentical	1 (4)	146 (45)
**Stem cells source**, n (%)		
Bone marrow	18 (70)	226 (70)
Cord blood	4 (15)	24 (7.4)
Peripheral blood stem cell	4 (15)	74 (23)
**Conditioning regimen**, n (%)		
Myeloablative	14 (54)	164 (51)
Reduced intensity	12 (46)	158 (49)
**Clinical grade of acute GvHD**, n (%)		
Mild (grade 1–2)	11 (42.4)	155 (48)
Severe (grade 3–4)	15 (57.6)	76 (52)
**Refractory acute GvHD**, n (%)	16 (62)	77 (24)
**Total**, n (%)	26 (100)	322 (100)

GvHD = graft-versus-host disease.

**TABLE 2.** Patients description

N°	Diagnosis	HSCT				Acute GvHD					Endoscopy		Viral infection		Outcome
		Age at SCT	Donor type	SC source	CR	Total clinical grade	Other involved organs	Main GI symptoms	GI clinical grade (at biopsy)	GI histological grade	Indication*	Type	PCR on MB	Second-line	Survival
1	ID (IPEX)	13	MRD	BM	NMA	3	-	Upper	2+	no GvHD	a	PC	neg.	no	alive
2	β-talassemia	5	MRD	BM	MAC	4	Skin	Lower	4+	1	b	EGD + PC	Adenovirus	yes	alive
3	AML	17	AD	BM	MAC	3	-	Upper	2+	no GvHD	a	PC	neg.	no	alive
4	AML	9	MRD	BM	MAC	4	Skin	Lower	4+	4	b	PC	neg.	yes	alive
5	Fanconi	9	AD	BM	NMA	3	Skin + liver	Lower	2+	1	b	PC	neg.	yes	alive
6	ALL	2	AD	BM	MAC	4	-	Lower	4+	1	a	FS/RSB	neg.	yes	alive
7	AML	15	AD	BM	MAC	3	-	Lower	4+	3	a	FS/RSB	neg.	no	dead
8	AML	5	AD	BM	MAC	4	Skin	Lower	4+	4	b	PC	neg.	yes	dead
9	AML	12	AD	BM	MAC	3	Skin	Upper and lower	4+	2	b	PC	CMV	yes	alive
10	CAMT	7	AD	CB	NMA	4	Skin + liver	Upper and lower	4+	4	b	FS/RSB	Adeno/HHv6/CMV	yes	dead
11	Fanconi	9	AD	PBSC	NMA	3	Skin	Lower	3+	1	b	PC	CMV	yes	alive
12	β-talassemia	14	MRD	BM	NMA	4	Skin	Lower	4+	1	b	PC	neg.	yes	dead
13	ALL	7	AD	BM	MAC	3	-	Lower	2+	1	a	EGD	neg.	no	alive
14	SAA	2	AD	BM	MAC	3	Skin	Lower	3+	4	b	FS/RSB	neg.	yes	alive
15	ALL	11	AD	BM	MAC	3	Skin	Lower	2+	no GvHD	b	FS/RSB	neg.	no	alive
16	SAA	14	AD	PBSC	NMA	4	Skin	Lower	4+	1	b	EGD + PC	neg..	yes	dead
17	ALL	11	AD	BM	MAC	4	Skin	Lower	4+	2	b	FS/RSB	neg.	no	dead
18	DC	9	AD	CB	NMA	4	Skin	Lower	3+	1	b	PC	neg.	yes	alive
19	AML	11	AD	BM	MAC	3	-	Lower	2+	1	a	FS/RSB	neg.	yes	alive
20	JMML	5	AD	BM	MAC	4	Skin + liver	Upper and lower	4+	no GvHD	b	EGD	neg.	yes	dead
21	ALL	1	AD	CB	NMA	3	-	Lower	2+	1	a	FS/RSB	HHV6	yes	dead
22	Fanconi	10	AD	BM	NMA	4	Skin + liver	Lower	4+	3	b	EGD + PC	EBV	yes	dead
23	MDS	14	MMRD	BM	NMA	3	Skin	Lower	2+	no GvHD	b	PC	neg.	yes	dead
24	AML	10	AD	PBSC	MAC	3	-	Upper	2+	no GvHD	a	EGD	neg.	no	alive
25	ID(Cernunnus)	10	AD	PBSC	NMA	3	-	Lower	2+	1	a	PC	neg.	no	alive
26	ID (FHL-5)	1	AD	CB	NMA	2	-	Lower	1+	no GvHD	a	EGD	neg.	no	alive

**AD** = alternative donor; **BM** = bone marrow; **CB** = cord blood; **CR** = conditioning regimen; **GI =** gastrointestinal; **GvHD =** graft-versus-host disease; **ID** = immunodeficiency; **IPEX** = immunodysregulation polyendocrinopathy enteropathy X-linked; **MB** = mucosal biopsy; **MRD** = matched related donor; **NMA** = non myeloablative conditioning regimen; **PBSC** = peripheral blood stem cell; **PC** = pan-colonoscopy; **PCR** = C reactive protein; **SC** = stem cell; **SCT** = stem cell transplantation.

*a = GI isolated GvHD at onset; b = partial response after first-line therapy of multi-organ a-GvHD because of persistent GI symptoms.

Ten children (38.5%) underwent endoscopy to confirm the suspicion of GI a-GvHD in the absence of any other organ involvement, while the other 16 (61.5%) underwent endoscopy for the persistence of GI symptoms after partial response to multiorgan a-GvHD therapy (Table 2). Endoscopic procedures were performed in the upper GI tract (EGD) in 4 children (15.4%), in the lower GI tract in 19 (73%) [PC in 11 (42.3%), FS in 3 (11.5%), and rectal suction biopsy in 5 (19.2%)], and combined upper and lower GI tract endoscopies (EGD + PC) in 3 (11.6%). At the time of histological evaluation, diarrhea was the most common GI symptom (84.6%). All mucosal biopsies, except in the case of rectal suction biopsy, were obtained under sedation. The median interval between allo-SCT and mucosal biopsies was 55.5 days (range 21–148) and that between clinical diagnosis of GI a-GvHD and mucosal biopsies was 23 days (range 1–103).

The clinical grades of GI a-GvHD calculated at the time of the endoscopic procedure were as follows: grade 1, one case (4%); grade 2, 10 (38%); grade 3, 3 (12%); and grade 4, 12 (46%). Histology confirmed the clinical diagnosis of GI a-GvHD in 19 patients (73.1%), while in 7 patients (26.9%) the histological findings were not specific and did not correlate with the clinical diagnosis of GI a-GvHD. Among the 19 patients with histological confirmation of a-GvHD, the histological grades calculated according to the Cruz-Correa criteria were as follows: grade I in 11 patients [5 patients (45.4%) with a GI a-GvHD clinical grade of 2, 2 (18.2%) with a grade of 3, and 4 (36.4%) with a grade of 4]; grade II and III in 4 patients (all with a a-GvHD clinical grade of 4); grade IV in 4 patients (three with a a-GvHD clinical grade of 4 and one with a clinical grade of 3). Viral PCR performed on mucosal samples was positive in 6 patients (23.1%), 5 of which were positive for a single virus (2 cytomegalovirus-CMV, 1 adenovirus, 1 Epstein Barr Virus-EBV, 1 herpes 6 virus-HHV6) and one was positive for three associated viruses (adenovirus plus CMV plus HHV6). Furthermore, in all these cases with viral infections, histological findings confirmed the presence of GI-a-GvHD. Macroscopic and histological assessments were graded identically in 66% and 86% of the lower and upper endoscopic procedures, respectively.

The sensitivity of histological evaluation was 57.1% (4/7) when the samples were obtained through upper endoscopies and 81.8% (18/22) when they were obtained through lower endoscopies.

Six of the seven patients without histological confirmation of GvHD had a clinical diagnosis of grade 1–2 GI-a-GvHD (85.7%) and one was grade 4 (14.3%); viral PCR on mucosal samples was negative. A high clinical grade (3 or 4) was more frequently associated with histological confirmation of GI a-GvHD than with a low clinical grade (1 or 2; *P* = 0.026).

GI a-GvHD was steroid-resistant in 15 (78.9%) of the 19 children with a histological diagnosis of GI-a-GvHD and in two (28.6%) of the seven patients with negative histology (*P* = 0.028). Among the patients with histological confirmation of GI-a-GvHD, histological grade was more frequent in patients with a high (3 or 4) clinical grade than in those with a low (1–2) clinical grade (*P* = 0.038).

At a median follow-up of 94.4 months after SCT (range 3.3–203.9 months), 10 patients died (38.4%): 2 with invasive mycosis, 3 with severe c-GvHD, and 5 with leukaemia relapse. The cumulative 3-year OS was 61.5% (95% CI, 40.3-77.1), while the 3-year TRM was 22.1% (95% CI, 9.8-45.3). The 3-year OS was 71.4% (95% CI, 25.8-92.0) in patients with negative histology and 57.9% (95% CI, 33.2-76.3) in those with histological confirmation of GI-a-GvHD (log rank *P* = 0.62). Moreover, the 3-year TRM was 0% in the group with negative histology and 28.9% (95% CI, 13.2-56.2) in patients with histological confirmation of a-GvHD.

One child who underwent combined GI endoscopy (EGD + PC) for symptoms affecting both upper and lower GI tract developed a duodenal intraparietal hematoma discovered one month later by abdominal X-ray and abdominal ultrasound (US), which were performed for suspected intestinal perforation or occlusion. Complete resolution of the hematoma was established by US evaluation 2 months later. No acute adverse events occurred after endoscopic procedures in any of the other cases.

## DISCUSSION

In our pediatric study, the sensitivity of histological evaluation in confirming the clinical diagnosis of a-GvHD was 73.1%, which was higher when endoscopy was performed in the lower GI tract (81.8%) than in the upper GI tract (57.1%), as reported in previous studies.

Histological confirmation of any grade of GI a-GvHD was associated with a significantly higher rate of steroid resistance (*P* = 0.028) and with an increased TRM (28.9%) compared with a negative histology. In contrast, patients with a high histological grade did not require second-line treatment more frequently than those with a low histological grade. Among the 19 patients with histological confirmation of a-GvHD, 8 (42.1%) had a histological grade lower than the clinical grade, wherein all received second-line therapy. The lack of correspondence between clinical and histological findings observed in some cases might be related to the patchy distribution of GvHD in the intestinal tract (9) and to steroid therapy if already ongoing at the time of the biopsy.

In our patients, the decision to perform the endoscopic evaluation of the upper, lower, or both GI tracts was made considering the symptoms presented by each patient and by the period of the study. The single adverse event we observed after a duodenal mucosal biopsy performed during an EGD in a patient with a high clinical grade of GI a-GvHD suggests that the endoscopic approach can be considered a safe procedure but not completely without risk. As reported by previous authors (12–15), it should be less invasive considering the risk of bleeding or intestinal perforation (16).

In our cohort, FS or rectal suction biopsy showed an equal or even higher sensitivity than PC and EGD, and it was not associated with any complications. In a study of 20 children who underwent both upper and lower endoscopy for the evaluation of a-GvHD, Crowell et al. (4) demonstrated that FS has the same diagnostic accuracy as EGD, is associated with a lower risk, and requires minimal sedation. Furthermore, duodenal biopsies may cause severe complications depending on the hematological conditions and tissue fragility in these young patients (6–8). These observations, confirmed by other authors (5,6), suggest that FS should be preferred, particularly in children, since it entails fewer risks and requires a lower procedural sedation while being equally effective in the diagnosis of GvHD compared with PC or EGD (17,18).

The main limitation of this study is its retrospective nature; gastrointestinal endoscopy was not performed in all patients with clinical suspicion or diagnosis of GI-a-GvHD, preventing us from defining its real sensitivity. On the other hand, this study offers the opportunity to address questions about the potential role of histological evaluation in the management of patients with clinical signs of GI a-GvHD in the absence of other organ involvement or with persistence of GI a-GvHD symptoms after response to therapy involving other organs.

Our study suggests that the endoscopic evaluation of children with a clinical suspicion of GI a-GvHD is a safe and feasible technique. Flexible sigmoidoscopy with rectal mucosa biopsy evaluation should be performed whenever possible because it is less invasive and provides similar information considering the potential procedure-associated risk. It should also be performed by pediatric gastroenterologists with proven expertise in this field.
